# Decoding the Misinformation-Legislation Pipeline: an analysis of Florida Medicaid and the current state of transgender healthcare

**DOI:** 10.5195/jmla.2023.1724

**Published:** 2023-10-02

**Authors:** KD Coldwater

**Affiliations:** 1 kd.coldwater@nau.edu, Health Science Librarian, Cline Library, Northern Arizona University, Flagstaff, AZ

**Keywords:** Transgender healthcare, critical librarianship, misinformation

## Abstract

**Background:**

The state of evidence-based transgender healthcare in the United States has been put at risk by the spread of misinformation harmful to transgender people. Health science librarians can alleviate the spread of misinformation by identifying and analyzing its flow through systems that affect access to healthcare.

**Discussion:**

The author developed the theory of the Misinformation - Legislation Pipeline by studying the flow of anti-transgender misinformation from online echo chambers through a peer-reviewed article and into policy enacted to ban medical treatments for transgender people in the state of Florida. The analysis is precluded with a literature review of currently accepted best practices in transgender healthcare, after which, the author analyzes the key report leveraged by Florida's Department of Health in its ban. A critical analysis of the report is followed by a secondary analysis of the key peer-reviewed article upon which the Florida Medicaid authors relied to make the decision. The paper culminates with a summation of the trajectory of anti-transgender misinformation.

**Conclusion:**

Misinformation plays a key role in producing legislation harmful to transgender people. Health science librarians have a role to play in identifying misinformation as it flows through the Misinformation - Legislation Pipeline and enacting key practices to identify, analyze, and oppose the spread of harmful misinformation.

## BACKGROUND

Health science librarians are often tasked with seeking out and delivering high quality health information [1]. As evidenced in the Medical Library Association (MLA) “Code of Ethics for Health Sciences Librarianship,” health science librarians serve “society, clients, and the institution” by ensuring that informed decisions can be made in the domains of health care, health science education, and health science or biomedical research [1]. Additionally, MLA's Core Values indicate a librarianship that excels in guiding the use of scientifically informed healthcare decisions, public awareness of quality health information, and “advancement of health information research and evidence-based practice (EBP)”, in particular [1]. In order to bring these values and ethical standards into practice, health science librarians also must be willing to understand how misinformation enters into health-based decision making, rhetoric concerning healthcare, and the means by which healthcare is governed at bureaucratic and legislative levels. This cyclical process can be termed the Misinformation – Legislation Pipeline. Understanding the function and consequences of the Pipeline is a necessary first step to dismantling it and upending its harmful effects, especially on minoritized communities.

In this paper, I will expose one such Misinformation - Legislation Pipeline, which helps sustain the disparagement and violent stigmatization of transgender communities in the United States. While there are many ways to “be trans” and express trans identity, for the purposes of this paper, the term transgender describes any person who harbors a gender identity that differs from the sex they were assigned at birth, including individuals who identify with a nonbinary gender identity. Additionally, it bears mention that many transgender people forego medicalization of their identity and may, therefore, opt out of one or any of the following medical and psychological treatments: gender affirming psychotherapy, puberty suppression, hormone replacement therapy, and gender affirming surgeries [2, 3]. At the same time, misinformation about transgender people still leads to health disparities for all transgender people [4]. That said, there continue to be a far greater number of transgender people who desire medical treatment than are able to procure it [3]. In the largest study yet published on transgender experiences in the United States, respondents indicated that people around them blocked access to medical treatment, with only 1% of respondents having received puberty suppressors at some point and while 91% reported a desire to procure some level of care, only 65% received care in any form whatsoever [3].

Although access to care has expanded over time, transgender people are now experiencing strict barriers to care, especially transgender persons of color, transgender children/adolescents, nonbinary persons, and transgender persons with disabilities [4 - 8]. In a 2015 survey of transgender adults, 55% of respondents were denied insurance coverage for gender-affirming surgery, 25% were denied coverage for hormone replacement, and 33% reported at least one negative interaction with a healthcare provider, including being put in a position to “teach” the provider about transgender healthcare (24%), being asked probing, invasive questions which were not warranted by the situation (15%), and being outright denied transition-related services (8%) [3]. Native American, Middle Eastern, Black, and Hispanic respondents all experienced more negative interactions and greater barriers to coverage [3].

For transgender children and adolescents, access to care is growing especially fraught and is increasingly beholden to a Misinformation - Legislation Pipeline that governs mental health services, puberty blocking medication, and hormone replacement therapy. Viewed through a historical lens, the use of 20th century and early 21st century medical gatekeeping has morphed into a tool whereby the state can use medical language and practice to place barriers around gender affirming care. In these cases, a child's home state functions as a social determinant of health. Children in states with trans-repressive medical regulations are put at a disadvantage even when they otherwise have access to gender-affirming providers and insurance coverage.

The growing number of health barriers experienced by transgender people stem from living in a society that is inherently transphobic. Transphobia can be defined in three manners. Firstly, “internalized transphobia” is comprised of shame, alienation from other transgender persons, and a harmful internalization of cisnormative gender expectations. The term “cis” is a Latinism which means “on this side”, and when used as a prefix in the term “cisgender”, designates individuals whose asserted gender identity is not in conflict with their sex assigned at birth. Additionally, “cisnormative” describes the ways in which the gendered actions of cisgender people are treated as normal, whereas the gendered actions of transgender, nonbinary, and intersex people are treated as abnormal. Second, “interpersonal transphobia” involves intentional or unintentional discrimination and victimization of transgender people by cisgender individuals or groups. Lastly, “structural transphobia” is a mode of structural oppression, which has been defined as “historically rooted cultural ideologies and interconnected institutions…[and is] unique from individual oppression because it is enacted through systems rather than just individuals with power and prejudice” [9, 10]. By recognizing that transphobia has a tripartite structure rooted in cisnormativity and structures which benefit cisgender people, it becomes clear that medical interventions on their own are not enough to resolve gender dysphoria (GD) and other trans health disparities [7]. Rather, society itself must change. While this means that many people from different disciplines can work together to establish a more health equitable society for trans people, it also means that bad faith actors can further systematize transphobia through toxic interpersonal discourse, the rapid spread of misinformation about transgender people, and ultimately, the enactment of legislation that criminalizes gender affirming healthcare (and by extension, transgender people).

Health science librarians are ideally positioned to insert ourselves within this aforementioned systematization of transphobia, namely by pinpointing misinformation, redirecting our library communities to established guidelines, collecting high quality information concerning gender affirming care, and sharing our expertise in public settings such as legislative sessions. However, it is also essential that we understand how this specific Misinformation - Legislation Pipeline develops and impacts transgender people. In doing so, we better prepare ourselves to identify the tactics deployed by purveyors of misinformation, the means by which anti-trans propaganda spreads, and how misinformation and propaganda lead to the enactment of harmful policies and legislation. For that reason, this article explores each stage of this process. First, the author will lay out current best practice, then describe the infusion of low quality research into the discourse, with a focus on Rapid Onset Gender Dysphoria (ROGD) as expressed within a Florida Medicaid policy document as a case subject. Next, the author identifies the throughlines that lead to the use of misinformation in lawmaking and concludes with a series of initial actions that health science librarians can practice in addition to a final statement about the political outlook concerning transgender healthcare in the year 2023.

### Current best practices in transgender healthcare

Transgender healthcare has existed in various forms for over a century [11, 12]. Magnus Hirschfield established the Institute for Sexual Research, the first modern clinic to serve transgender people in 1919 [12, 13]. At the same institute, patient Dora Richter received the first known gender confirmation surgery in 1931 [12, 13]. In the United States, Johns Hopkins University became the first American school to open a medical program focusing on transgender medicine and research [12, 14]. The Johns Hopkins program was ended in 1979, but in the same year, the World Professional Association for Transgender Health (WPATH), one of the most important organizations for researching and guiding transgender healthcare, was developed by endocrinologist Harry Benjamin [15].

Since that time, WPATH has published 8 iterations of the WPATH Standards of Care. The current edition, SOC8, was published in 2022, and is the collaborative work of 119 authors spanning endocrinological, laryngological, pediatric, psychiatric, psychological, public health, reproductive, and surgical disciplines. SOC8 provides practice guidelines that extend across the lifespan and also take global and intersectional medical needs into account. Building on SOC7, the current edition also includes guidelines for the care of nonbinary and intersex individuals. Regarding pediatric care, SOC8 provides guidance for “the use of puberty suppression and, when indicated, the use of gender-affirming hormones” [16]. Further recommendations include long term care by professional medical teams to determine surgical needs as adolescents near adulthood [16]. Importantly, SOC8 also makes clear that gender affirming care must function through a biopsychosocial framework that takes into account various modes of transphobia and social as well as psychotherapeutic solutions that function alongside medical treatment. The SOC8 is widely available, and is free to use and download through the WPATH website at: wpath.org/publications/soc.

In addition to the WPATH SOC8, health science librarians ought also to be familiar with guidelines and statements produced by professional medical and healthcare organizations in the United States. Of note, in 2018, the American Academy of Pediatrics (AAP) published a policy statement titled, “Ensuring Comprehensive Care and Support for Transgender and Gender-Diverse Children and Adolescents”. In addition to ensuring social and psychological wellbeing, the AAP also stresses the value and necessity of medical management, including pubertal suppression and hormone replacement at the appropriate ages and in the appropriate conditions [17]. Likewise, in 2018, the American Academy of Family Physicians (AAFP) published guidelines for primary care providers titled, “Caring for Transgender and Gender Diverse Persons: What Clinicians Should Know”. Recommendations include cultural sensitivity trainings, routine mental health screens, hormone replacement therapy as needed, and pubertal suppression as needed [18]. Like WPATH and AAP, the AAFP guidelines explicitly discourage the use of “conversion therapy”, which describes pseudoscientific attempts to revert a transgender person's internal gender to match their sex assigned at birth [16 - 18]. In 2017, The Endocrine Society (TES) also published guidelines on transgender healthcare with recommendations for endocrinologists in the United States. TES re-endorsed its guidelines in a follow-up position statement in 2020. The guideline, titled, “Endocrine Treatment of Gender-Dysphoric/Gender-Incongruent Persons: An Endocrine Society* Clinical Practice Guideline” provides recommendations for pubertal suppression and hormone replacement in adolescents and adults as well as recommendations for treating transgender patients undergoing gender affirming surgeries both pre- and post-op [19]. The American Medical Association (AMA) has not published guidelines for practice; however, in 2021, the AMA issued a letter to the National Governors Association which states:

**Empirical evidence has demonstrated that trans and non-binary gender identities are normal variations of human identity and expression. For gender diverse individuals, standards of care and accepted medically necessary services that affirm gender or treat gender dysphoria may include mental health counseling, non-medical social transition, gender-affirming hormone therapy, and/or gender-affirming surgeries. Clinical guidelines established by professional medical organizations for the care of minors promote these supportive interventions based on the current evidence and that enable young people to explore and live the gender that they choose. Every major medical association in the United States recognizes the medical necessity of transition-related care for improving the physical and mental health of transgender people [20].**

Taken together, the slew of guidelines, standards of care, and position statements published by professional organizations suggests a near-unanimous recognition that transgender affirming healthcare should at least ascribe to the following criteria:

- initiate non-surgical and non-hormonal social changes that affirm gender identity;
- initiate pubertal suppression as needed and under the correct circumstances;
- initiate hormone replacement therapy as needed and under the correct circumstances;
- support or provide gender confirming surgeries as needed and under correct circumstances.

## DISCUSSION

### Misinformation: A Florida Case Study

With this near-unanimous support by US healthcare guidelines, it is worth considering why the Florida Department of Health (FDH) published a statement on the “Treatment of Gender Dysphoria for Children and Adolescents” in 2022 which provides recommendations that are entirely contrary to those mentioned above. Stated in direct quotations: “Social gender transition should not be a treatment option for children or adolescents. Anyone under 18 should not be prescribed puberty blockers or hormone therapy. Gender reassignment surgery should not be a treatment option for children or adolescents” [21].

In support of these guidelines, the FDH cites a single 2021 opinion piece published by psychologist David Schwartz in the *Journal of Infant, Child, and Adolescent Psychotherapy* as “currently available evidence.” Schwartz's article contains no verifiable, original research outside his perspective and history as a child psychologist [22].

Upon publication of the FDH guidelines, Florida Medicaid also published the document “Generally Accepted Professional Medical Standards Determination on the Treatment of Gender Dysphoria” and simultaneously ended coverage of necessary gender affirming healthcare for all individuals, including adults [23]. The Medicaid decision was approved under the auspice that evidence for treatment outcomes is “weak to very weak” without any randomized control trials (RCTs) to support the use of gender affirming treatment (it bears mention that a RCT performed on any transgender population which involves hormonal treatment, pubertal suppression, or gender confirming surgeries would be ethically untenable and logistically challenging, as it would place undue burden on participants in a control group) [23]. In December 2022, the Florida Board of Medicine and Florida Board of Osteopathic Medicine moved to ban the medical treatment of anyone under the age of 18 for GD [23].

Considering the overt contradictions between the FDH guidelines and the aforementioned guidelines from the WPATH, AAP, AAFP, AMA, and Endocrine Society, it is necessary to consider how multiple medical organizations in Florida came to the conclusion that gender affirming healthcare should be unfunded for adults and banned for children and adolescents. FDH and Florida Medicaid's decision making relies on the findings presented in the aforementioned Florida Medicaid report. This report presents a rationale for the undoing of gender affirming healthcare based on a wide range of first and secondary sources [23]. In doing so, the authors flatten the concept of evidence-based practice to include findings from non-scholarly resources such as NBC News, Fox News, Tampa Bay Times, a student essay posted to the blog Students 4 Best Evidence, and The New York Times. While including news stories and non-peer reviewed work in medical decision making is not inherently flawed, it does call into question the purpose of Florida Medicaid's guiding document and whether it purports to adhere to generally accepted standards of evidence-based practice. In total, the document relies on 88 citations, 27 of which are primary sources detailing original research. Four of these 27 citations only appear in the Works Cited and are not linked to any citations in the document. Additionally, the authors misrepresent or misinterpret the findings of another four of the 27 published studies. Otherwise, of 88 total citations, the authors rely on the published findings of: 27 original research articles (four missing from body of paper), 25 reviews (including literature reviews, statements and organizational reports), 20 opinion articles (including letters to the editor, comments, and bioethical arguments), 6 news articles, 5 encyclopedia entries, 4 articles labeled as “unpublished” (and which were irretrievable), and 1 conference proceeding.

Therefore, including the eight questionable citations, 31% of cited sources convey some level of original research. Also of note: two systematic reviews fall in this category; however, one (Costa et al, 2016) only appears in the Works Cited and not in the body of the text. The second (Davy & Toze, 2018), “What is Gender Dysphoria? A Critical Systematic Narrative Review” was published in The Journal of Transgender Health in 2018. Davy & Toze (2018) focus on the proliferation of definitions and descriptions that encapsulate the term “gender dysphoria”, as well as the problematic ways in which it is leveraged as a medical term via the “Diagnostic and Statistical Manual of Mental Disorders 5” (DSM-V) and via usage of the term GD in social discourse in ways that further distance GD from the lived experiences of transgender persons [24]. Florida Medicaid (2023) recognizes their research goals but appears to misinterpret their findings, specifically by inferring that Davy and Toze (2018) are concerned with individuals being misdiagnosed with GD, which “raises the question of whether individuals are receiving potentially irreversible treatments…” [23]. To the contrary, Davy and Toze express concern that individuals aren't receiving the gender-affirming care that they need because they do not fall within the strict limitations set by DSM-V [24].

Aside from the possible misappropriation of Davy and Toze's research, Florida Medicaid relies heavily on one other original research article. This paper, “Rapid-Onset Gender Dysphoria in Adolescents and Young Adults: A Study of Parental Reports”, is more heavily sourced than any other research article in the document, being cited six times throughout. Published by Lisa Littman in 2018 in the journal, PLOS One, “Rapid-Onset Gender Dysphoria in Adolescents and Young Adults: A Study of Parental Reports” describes a phenomenon that Littman describes as “rapid onset gender dysphoria” (ROGD), a subset of GD that involves a sudden interest in gender expression and gender identity among children and adolescents, generally whom were assigned female at birth (AFAB) [25].

Outside of Davy and Toze (2018), Littman's study is included as the sole evidence to justify the delay and/or eradication of care for transgender children and adolescents. This is reason enough to warrant an analysis of Littman's study. As a key piece of evidence that informs Florida Medicaid's decision making, readers may presume that this paper presents authoritative research which calls into question the practice rationales by WPATH and other professional organizations. According to Florida Medicaid, Littman (2018) is one of multiple studies to introduce “additional subtypes of gender dysphoria” (in this case, ROGD) [23]. It is not clear which studies outside of Littman's 2018 article do propose additional subtypes, as–at this time–no such studies exist in the peer reviewed literature outside letters to the editor, specifically by Sinai et al (2022), Littman (2022), and Hutchinson et al (2020) [26 - 28]. Rather, the only published original research that follows up on Littman's 2018 study on ROGD “…did not find support within a clinical population for a new etiologic phenomenon of rapid onset gender dysphoria during adolescence” [29]. In other words, the existence of ROGD is unsupported in current research.

**Figure 1:**
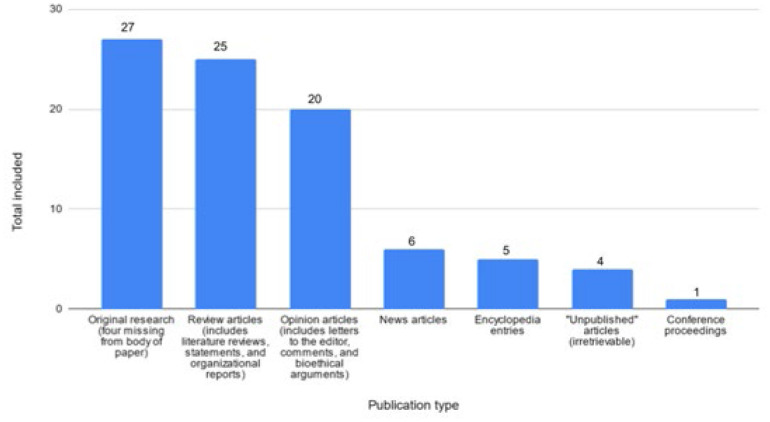
Cited publications by type

### The Misinformation – Legislation Pipeline

While ROGD has not been identified in research literature, it has exploded across the discourse concerning gender affirming healthcare for adolescents. For that reason, cursory knowledge about Littman's paper is warranted. Littman came to her conclusion that ROGD is a subcategory of GD through an exploratory study consisting of a survey distributed to parents of transgender children culled from three online forums: 4thwavenow, TransgenderTrend, and youthtranscriticalprofessionals. Based on participant responses, Littman found that parents identified the following changes in their children: sudden declines in mental health, self-isolation, mistrust of family members, “only trusting information about gender dysphoria from transgender sources”, increased engagement in social media, and having friends who identified as transgender [25]. While Florida Medicaid treats these findings as key evidence, there are noticeable problems with the article. First and foremost is Littman's treatment of trans identity as pathological or diseased. As Restar (2020) argues, “the majority of methodological and design issues stem from the use of a pathologizing framework and language of pathology to conceive, describe, and theorize the phenomenon [of ROGD] as tantamount to both an infectious disease…and a disorder” [30]. In framing GD as a disease, Littman perpetuates the structurally transphobic medicalization of transgender people. She frames being trans as something that ought to be corrected. In her article, more young people identifying as trans is not indicative of a more accepting and tolerant society. Rather, more trans children indicates a transmittable disease, or a “social contagion” in both her own words and in the words of Florida Medicaid [23, 25]. Littman does attempt to distance her use of social contagion from its harmful connotations, but at the same time, she opts to compare the increasing number of trans children to the increase in children with eating disorders, two entirely different social phenomena [25].

In addition to misconstruing GD as a disease, Littman heavily relies on convenience sampling by opting to survey participants from similar demographic backgrounds (white, middle aged, ciswomen with college educations). Additionally, the participants were already suspicious of transgender identity prior to inclusion in Littman's study. As Restar (2020) points out, 76.5% of respondents “believed that their child's trans identification is not correct” [30]. And critically, all of Littman's participants were sampled from populations on three online forums that have earned notoriety for pushing anti-transgender misinformation and in time, have aligned with “trans-exclusionary reactionary” (TER) and “gender critical” movements (TER/GC) [30]. Since participants were active in TER/GC spaces, Littman's conclusions concerning ROGD are at the very least non-representative of wider trans experience and may constitute confirmation bias.

TER is a term used primarily in transgender communities and among allies to describe individuals who adopt anti-trans political and ideological views. GC is a self-identifying term used by people who hold a spectrum of anti-trans views. For the purpose of this paper, the two acronyms are coalesced as TER/GC. Many TER/GC communities have formed in isolated, small networks such as 4thwavenow and TransgenderTrend. This is partially due to holding views that are considered discriminatory and which break the End User License Agreements on most prominent social media platforms. For instance, r/gendercritical, one of the larger TER/GC online communities, was banned by Reddit in 2020 for breaking rules concerning transphobia (Tiffany 2020). As reported by Kaitlyn Tiffany (2020), groups receiving bans and closures have opted to build insulated platforms, including 4thwavenow, Mumsnet, Ovarit, TransgenderTrend, and #radfem [31]. In doing so, TER/GC individuals establish echo chambers that aid the flow of transphobic misinformation. Online echo chambers are key to propagating extremist ideologies, constructing false beliefs, and finding gaps wherein misinformation can be injected into wider discourses [32, 33].

TER/GC echo chambers operate as the frontline in establishing misinformation that harms transgender people. They are tied to ideas that transwomen are co-opting “womanhood”, that opening public bathrooms to transgender people will result in greater sexual violence towards children and cisgender women, that transwomen are prone to violent behavior, that transgender people are delusional, and that transgender people are “grooming” children for the purpose of sexualizing them–an idea that closely adheres to the idea that “ROGD” is driven by social media influencers and celebrities that make being transgender look trendy [34 - 36]. From these (misinformed) positions, further misinformation is likely to develop, thus constituting a cycle wherein information feeds in on itself and propagates more information while strengthening the crystallization of misinformed beliefs. For instance, although transgender people are more likely than cisgender people to experience violence in bathrooms, TER/GC sympathizers have called for policies that would result in segregation of transgender people from gender affirming bathrooms, locker rooms, and other sex-specific facilities [3]. At the extreme end of the misinformation spectrum, calls for the institutionalization and eradication of transgender people are common attributes of these echo chambers, though often, they are couched in plausible deniability and calls for political action that would subjugate transgender populations [34]. Unsurprisingly, these echo chambers provided fertile ground for Littman to develop the concept of ROGD, an unsupported subdiagnosis of GD that relies on pathologization and misinformed beliefs about transgender people, and which ultimately, lends an air of scientific credibility to TER/GC ideology. In migrating out of echo chambers and into peer-reviewed research streams, anti-trans misinformation can then be spread more widely. This makes up the second phase of the Transgender Healthcare Misinformation – Legislation Pipeline, when misinformation moves out of insulated spaces into credible channels.

Having described ROGD and its place in the first two stages of the Misinformation – Legislation Pipeline concerning transgender healthcare, it is worth returning to Florida Medicaid's use of Littman's article as a key document in its decision to regulate transgender healthcare. Since publication of Littman 2018, findings of ROGD have not been replicated. On the contrary, Bauer et al. found that ROGD as a social phenomenon is not supported by clinical data provided by transgender adolescents [29]. As Bauer et al. (2022) point out, data provided by transgender children might come into conflict with parents' perspectives, not due to contact with “peer contagion”, but due to parents adopting transphobic and cisnormative beliefs and actions, which result in their children opting to hide or downplay any GD [29]. In contrast, research consistently shows that children supported by parents in their trans identities are more likely to have lower rates of mental illness than children who are not [37 - 39]. At this time, ROGD has not been identified outside the bounds of Littman 2018, and in the case of Bauer et al (2022), there is research questioning the veracity of ROGD as an actual phenomenon. Even so, Florida Medicaid sidesteps its own claims that RCTs ought to be treated as gold standard in evidence-based decision making by dedicating more language to ROGD as evidenced in Littman 2018, a study derived from survey data, than to any other concept and relies on ROGD as high level evidence in its decision making process. As previously noted, rigorous, peer-reviewed studies in support of transgender healthcare are universally handwaved by Florida Medicaid as “unreliable” due to the lack of studies involving randomization and control groups. While RCTs are considered gold standard in some cases, they are not always optimal, nor are they necessary to procure best evidence for care [40].

Even though scholarly research has yet to support the existence of ROGD, the concept itself has proliferated far beyond Florida Medicaid. It is arguable that the three years in which cultural awareness of ROGD extended beyond Littman 2018 may be a key factor in its robust inclusion in Florida Medicaid (2023). Since publication, Littman 2018 has received 487,243 views in PLOS One, making it the 24th most viewed article (out of 284,722 total articles) in the PLOS One database (based on data collected in July 2023) [25]. However, its count of 173 total citations (in articles published in scholarly, peer-reviewed journals) suggests a wide gap between consumer clicks and critical, academic engagement with the content. Additionally, an Altmetrics search indicates that Littman 2018 has been cited by 3,220 users on Twitter with a total of 5,625 tweets reaching an upward bound of 16,965,366 followers.

News publications have assisted in further amplifying Littman 2018, and an Altmetrics search of most recent news articles indicates that it has been covered in center-right and right-leaning news outlets, including: the New York Post (“Liberal media refuses to tell the truth about transgender kids”), The Epoch Times (“Parents opposing child gender reassignment procedures are wrongly threatened”), The Daily Caller (“Growing evidence shows ‘transgender’ movement driven by ‘peer influence’), National Catholic Register (“With parental rights in spotlight on election day, some Christian teachers balk at using gender pronouns”), Newsweek (“How can we explain rising gender dysphoria among girls?”), and Brietbart (“Ann Coulter: teen girl enthusiasms–twitching, cutting, and trans”). Note that the political lean of these sources was derived from Ground. News, Media Bias / Fact Check, and AllSides [41 – 43].

These data indicate that this article has widespread appeal extending far beyond its applicability within medical literature. Additionally, platforming Littman 2018 in news publications has served to establish the notion that ROGD is a scientific, verifiable phenomenon, and one which “explains” increased numbers of young people identifying as trans. Even though the aforementioned publications generally lean conservative, they need only assert the veracity of Littman 2018 and ROGD in order to move political conversation such that ROGD is no longer a matter of research inquiry, but instead is one “side” of a debate about transgender people. In doing so, they indicate the third phase of the Transgender Healthcare Misinformation – Legislation Pipeline, where misinformation enters mainstream discourse.

ROGD has now spread outside the bounds of medical literature. It has a specific tag in the TER/GC websites TransgenderTrend and 4thwavenow (two of the sites from which Littman culled participants for Littman 2018). Further, A search of “ROGD” in TER/GC message board Mumsnet returns 2,528 results. ROGD is also treated as a rationale for the transgender “craze” covered by Abigail Shrier in the popular TER/GC book, Irreversible Damage [44]. In a move that foreshadows the methods employed by Florida Medicaid, Shrier dedicates the second chapter of her book to ROGD, relies on Littman 2018 as a foundational text for the entirety of Irreversible Damage, and accepts Littman's hypothesis and findings without question, treating them as solid evidence [44]. Irreversible Damage is a solidly TER/GC text with anti-trans leanings. Shrier herself says as much:

**“I wonder things I don't say aloud, too: Whether this transgender craze isn't partially the result of over-parented, coddled kids desperate to stake out territory for rebellion. Whether is no coincidence that so many of these kids come from upper-middle-class white families, seeking cover in a minority identity? Or is it the fact that they overwhelmingly come from progressive families–raised with few walls, they hunt for barriers to knock down?”**

[44]

These hypotheses are misinformative. None are affirmed by academic literature. To the contrary, the number of transgender children is evenly spread across the United States, with the greatest number of known trans youth living in the American South [45]. Additionally, the racial and ethnic makeup of transgender youth is mostly in line with those of the general population; however, a UCLA Williams Institute report has found that a larger number identify as Hispanic, while the number of white, transgender youth is lower than that of the white, cisgender population [42]. Vitally, transgender youth and transgender people as a whole are more likely to experience low-income status than the general population, and 40% of transgender youth experience mental illness leading to suicidal ideation and attempts prior to the age of 18 [3]. Contrary to positions staked out by individuals such as Littman and Shrier, and in contrast to information presented in Florida Medicaid, transgender youth experience a wide range of health and healthcare disparities [3]. They are more likely to experience lack of social support, homelessness, domestic violence, educational violence, and police violence [3, 46]. They have elevated rates of mental illness, including depression, anxiety, suicidal ideation, and substance use disorders [3, 43]. They are more at risk of contracting sexually transmitted infections, including HIV [3, 43]. At the same time, transgender people are more likely to have reduced GD and lowered rates of comorbid mental illness when they have access to gender affirming healthcare interventions, accepting social and family environments, and greater access to participate in social life [7, 47 - 50].

Unfortunately, the Misinformation - Legislation Pipeline provides a means for transphobic political actors to gloss over medical research about transgender healthcare while perpetuating misinformative notions like ROGD to expand and result in legislation that cuts off transgender youth from the services and medical support that are shown to alleviate the disparities they face. In 2022, 26 anti-trans bills were passed in 13 states [51]. In Alabama, Arizona, and Louisiana, bills were passed that outlaw gender-affirming healthcare in some form in 2022 [51]. As of Summer 2023, the number has ballooned to 541 anti-trans bills introduced across 49 US states. Thus far, 52 have been signed into law, 18 of which are specific to healthcare [51]; although, it is worth remembering that transgender health disparities arise from internal, interpersonal and structural transphobia, which are entrenched not only in healthcare but across education, employment, policing, housing, and other systems. Additionally, Florida Medicaid functions as a reminder that anti-trans regulations can be passed into legal and regulatory systems without the need for legislation, instead relying solely on bureaucratic levers such as social welfare systems (like Florida Medicaid) as well as public health, funding, and research arms (like the Florida Department of Health). Regardless of the method, political maneuvers such as these make up the fourth phase of the Transgender Healthcare Misinformation – Legislation Pipeline, as pictured above.

**Figure 2:**
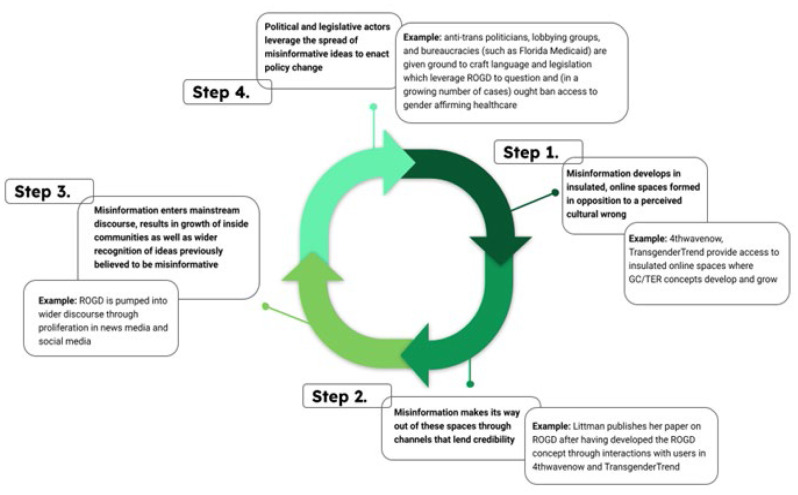
The Misinformation – Legislation Pipeline

Note that these four stages are not linear; instead, they often function in tandem with one another, producing and reproducing both misinformation and propaganda. Additionally, the process is not complete when the fourth stage is reached. Rather, the process is likely to continue cycling forward, as communities of misinformation creators develop novel means of legislating an assumed harm out of existence; in this case, that is transgender people.

## CONCLUSION

### Librarians' Responsibility

As health information experts, health science librarians have a role to play in understanding and calling attention to Misinformation - Legislation Pipelines. As evidenced by the progression of ROGD from Littman 2018 and into policy documents such as Florida Medicaid, misinformation carries the potential to spread from point of origin through social systems and into legal/political apparatuses that result in material harm to minoritized populations. In applying a critical lens to adverse policy documents, health science librarians can help uncover the means by which the authors of such documents rely on misinformation and/or propaganda to lend the appearance of credibility to harmful policies. By applying this analytical framing involving one specific policy document (Florida Medicaid) and ROGD as a concept, health science librarians have a blueprint that can be followed to perform further research and take a more active role in ensuring that misinformation which perpetuates health and healthcare disparities is both called out and critically analyzed.

Librarians should ensure that collections do not feed Misinformation - Legislation Pipelines. This includes print and electronic resource collections, even though it is more difficult to monitor the veracity of e-resources to the extent that print resources can be monitored. To better ensure the quality of collections, consider performing a DEI audit [52]. Collections should be assessed by expert librarians alongside trans-informed advisory committees for their capacity to represent a diverse array of voices, especially from people belonging to minoritized populations [52]. Furthermore, titles that perpetuate misinformation should be reclassified, placed on request, or withdrawn. While it is tempting to retain titles for purposes of remaining “neutral”, it is important to consider that neutrality language is illusionary, that information is never value free, and that neutrality continues to be used as a weapon to craft policies that harm minoritized communities [53]. Focus on developing HS Library collections to center academic, scholarly research that has been well vetted by librarians with subject expertise alongside advisories representing minoritized groups.

Librarians should also be willing to redirect students and researchers away from misinformation and challenge inclinations that “both sides” of a politicized debate must always be included for research and/or learning purposes. Instead, librarians should consider making use of misinformative material to provide learning opportunities for students. This includes sharing instances of Misinformation – Legislation Pipelines with students in instructional settings and to faculty in regard to curriculum design. As an example, students can be tasked with reviewing and critically analyzing the information conveyed in the American Academy of Pediatrics' literature on healthcare for trans children compared to the misinformation perpetuated on the website of the lobbying group, the “American College of Pediatricians”, which has been classified as a “fringe, anti-LGBTQ hate group” by the Southern Poverty Law Center [54]. Further, librarians should design learning materials that provide students with introductory knowledge about misinformation in various forms, whether it appears as propaganda, half-truth, outdated research, or unverifiable research.

From a more advanced perspective, an Evidence-Based Practice class can involve students pulling citations from legislative documents directly related to contested aspects of healthcare. Students can outline the number of citations and the publication type for each in order to procure a better understanding of evidence and its use in determining legislation and policy. Examples include healthcare related to environmental racism, racism in clinical contexts, transgender healthcare, healthcare reform, and reproductive health [55 - 59].

Librarians should also learn how lobbying groups, private research institutes, and political parties make use of misinformative sources in order to further political goals. As an example for how behind-the-scenes groups function and the subsequent consequences, read the journalist Madison Pauly's report in “Mother Jones” on March 8, 2023, titled “Inside the Secret Working Group that Helped Push Anti-Trans Laws Across the Country”. Pauly's reporting provides insight into the process of writing anti-trans legislation and the rapid spread of that “model legislation” by way of organizations like the Alliance Defending Freedom (ADF) and the aforementioned American College of Pediatricians (ACP) [60]. Groups like the ADF and the ACP play a vital role in the perpetuation of Misinformation – Legislation Pipelines, and as such, it is imperative that students, faculty, researchers, and clinical partners are aware of them. Additionally, professional library organizations, libraries, and individual librarians ought to move beyond the classroom or library itself and take part in activism and organizing efforts. Possibilities include the development of committees that meet directly with politicians, cultivating political relationships at local and regional levels, and promoting speaking efforts at legislative sessions.

Bearing in mind all four stages of the Transgender Healthcare Misinformation – Legislation Pipeline and the violent harm being done to transgender people in the United States through policy and the oppressive regulation of transgender healthcare, health science librarians should recognize that we have a responsibility and a capacity to affect change. Health science librarians are professionally situated to critically analyze legislation, policy, and other forms of regulatory writing, in order to ensure that misinformation and propaganda are not being used as leverage to stifle the provision of healthcare or prevent the enactment of equity, socially just care practices for transgender and other minoritized populations.
